# ‘Tis in our nature: taking the human-cannabis relationship seriously in health science and public policy

**DOI:** 10.3389/fpsyt.2013.00006

**Published:** 2013-02-26

**Authors:** Sunil K. Aggarwal

**Affiliations:** Department of Rehabilitation Medicine, New York University School of MedicineNew York, NY, USA

To find clearheaded scientific perspective on cannabis use through the prevailing thick smokescreen requires recognizing just what sort of smoke obscures our better understanding. In the United States, in large part, the smokescreen is made up of culture war-charged political rhetoric and obstructionism from those in positions of authority setting up a prejudicial ideological framing for cannabis use. National leaders throughout the twentieth century have taken opportunities afforded by high office or its pursuit to publicly opine on the dangers of cannabis, such as when then-Presidential candidate Ronald Reagan famously stated in 1980 that “leading medical researchers are coming to the conclusion that marijuana, pot, grass, whatever you want to call it, is probably the most dangerous drug in the United States and we haven't begun to find out all of the ill-effects. But they are permanent ill-effects. The loss of memory, for example Grass (1999).” Not only is such rhetoric overly simplistic, it also obscures and distorts pre-existing facts. In this particular case, Reagan's statement obscures the fact that the American Medical Association testified in 1937 on record to Congress that, after nearly 100 years of professional experience in Western medical practice with over 2000 prescribable marketed cannabis preparations (Antique Cannabis Museum, 2012), practitioners found that cannabis had an irreplaceable therapeutic role as an aid in the *remembering of old and long-forgotten memories* in psychotherapy patients (U.S. Congress, 1937). When in office, Reagan's first drug czar, Carlton Turner, blamed cannabis use for young people's involvement in “anti-military, anti-nuclear power, anti-big business, anti-authority demonstrations” (Schlosser, 1997), all dissenting positions toward government initiatives. Such clear scapegoating rhetoric has roots in the government's racialized Reefer Madness campaign of the 1930s which linked cannabis use in Blacks, Latinos, jazz musicians, and juvenile delinquents to racial miscegenation and homicidal mania (Helmer, 1975).

With such a long tradition of distorting rhetoric emanating from leading political authorities and being broadcast widely by the mass media, it is apparent how politicized cannabis use has become and how scientific research and knowledge about its use have been selectively highlighted and skewed to support pre-determined political objectives. These persistent distortions and political evasions are the greatest contributors to the smokescreen that obscures collection and dissemination of accurate evidence on cannabis use. The smokescreen is perpetuated because, as the saying goes, in war, the first casualty is the truth. Maintaining existing controversial policies relegating cannabis to the status of contraband (such as, under US federal law: zero-tolerance for use, a death penalty for trafficking amounts greater than approximately 66 tons, and official denial of currently accepted medical use in treatment) tends to be of a greater priority to governmental bodies than collecting and collating basic evidence regarding its use to inform public policy and health.

What evidence is gathered is often rejected or simply ignored if politically inexpedient. Here are a few examples. On occasion, political leaders are actually caught attempting to make “backroom” deals to ensure that a scientific commission's findings on cannabis use will have a predetermined outcome intended to marginalize political enemies. Take, for example, what was explicitly caught on tape during Richard Nixon's presidency. As documented on declassified tape recordings from the White House Oval Office on September 9, 1971, Nixon privately told his appointed Commission chair, former Pennsylvania Governor Raymond Shafer, that it was “terribly important” the Commission, tasked by Congress with helping to determine what level of risk cannabis use should be understood to constitute for the purposes of legal regulation, not come out with a report that was “soft on marijuana.” Strategizing for political expediency over factual review and nuance, Nixon called for obfuscation: “I think there's a need to come out with a report that is totally, uh, uh, oblivious to some obvious, uh, differences between marijuana and other drugs, other dangerous drugs… ” Nixon further warned Shafer: “Keep your Commission in line (CSDP, 2012).” Despite the Commission's recommendations to the contrary, cannabis was nevertheless maintained in the most restrictive category under federal law, Schedule I, where it has remained alongside heroin for 42 years, officially deemed to be devoid of medical utility, or safety. After a 14-year-delayed evidentiary hearing on a citizen-led cannabis-rescheduling petition filed in 1972 which lasted for 2 years, a Drug Enforcement Administration (DEA) Administrative Law Judge (ALJ) ruled in 1988 that cannabis should be rescheduled to Schedule II, with painkillers and anesthetics such as morphine and cocaine with currently accepted medical uses, and that to not do so would be “unreasonable, arbitrary, and capricious (SLDP, 2012).” The presidentially-appointed head of DEA rejected his own agency judge's ruling and, in 1994, a federal court finally denied the petitioners' appeal. An additional citizen-petition to reschedule cannabis filed in 2002 was rejected by the DEA after 9 years of delay and is presently under appeal (ASA, 2012). In 2007, another DEA ALJ ruled that it would be “in the public interest” to have more than one licensed facility to produce research-grade cannabis, and that a Plant and Soil Sciences Professor petitioner who had applied in 2001 for a production license and been denied be granted one. This DEA judge's ruling, too, was rejected by the DEA head in 2009 and is presently under appeal (MAPS, 2012). The rejection had the effect of allowing the federal government's hamstringing of scientific research to continue, with cannabis clinical studies being approved at an unacceptably slow pace, testing substandard-quality material produced under a government-backed private monopoly, and supplied only after potential investigators have waded through tremendous red tape, if supplied at all. Meanwhile, over the same timeframe, private pharmaceutical interests backed by highly-profitable international corporate pharmaceutical distributors have been granted license by the DEA to import and test in large, multicenter clinical trials in the US proprietary whole plant cannabis extracts made in company-owned cannabis production greenhouses licensed by friendlier governments (Aggarwal, 2010).

The persisting Schedule I classification of cannabis that the federal government maintains is itself a smokescreen that is directly discordant with authoritative, independent, medico-scientific evidence-based assessments. Publishing in the open-access scientific literature housed in the U.S. National Institutes of Health's National Library of Medicine, clinical investigators who oversaw seven separate, government-authorized, gold-standard design clinical trials of the safety and efficacy of smoked and vaporized inhaled cannabis for specific indications conducted at University of California medical centers over a 10 years period from 2002–2012 involving over 300 human subjects reported in an article entitled “Medical Marijuana: Clearing Away the Smoke” that all trials independently showed benefit. The authors concluded that the Schedule I classification of cannabis, based on the evidence collected and reviewed, is “not tenable,” “not accurate,” and one of the main “obstacles to medical progress (Grant et al., 2012).” This position is concordant with the analyses and conclusions in evidence-based positions papers and reports on cannabis medical science from leading national medical academies and specialty societies (National Research Council, 1999; American College of Physicians, 2008; American Medical Association, 2009).

To begin to clear such a thick and recalcitrant smokescreen of political rhetoric and interference surrounding cannabis use requires that a massive gust of fresh air be let into the room. This will help to spur a fundamental perspectival reorientation that will allow us to breathe freely, return to first principles, and start evidence-gathering from the beginning. An expedient smokescreen clearing approach is a historical and comparative ecological one that focuses on the human-cannabis relationship on a species to species level. We will come back to the theoretical outlines of this approach; for now, consider its results. While Cannabis sativa evolved in the Central Asian-Himalayan region ~36 million years ago (McPartland and Guy, 2004), it has spread to all regions of human habitation due to the long-standing fondness Homo sapiens have had for this semi-domesticated botanical cultivar, evidenced by the undisputed prehistoric archaeological record (Russo et al., 2008) and ancient textual references (Hillig, 2005). Cannabis's very name belies its longstanding relationship with humanity, as it was pragmatically given the species name “Sativa” in 1542 by German physician-botanist Leonhart Fuchs, meaning “cultivated” or “useful” in Latin (Russo, 2007). It grows easily in numerous climates as a wild and hardy plant whose palmate fan leaf's geometry is iconic. Uses of Cannabis sativa include production of textiles, building material, canvas, rope, paper, and biofuel using the cellulose and fiber of its stalk; nutritive food, edible oil, and lotions using its oil- and protein-rich seeds; and, most pointedly, herbal medicines, spiritual sacraments, and psychoactive inebriants using its phytocannabinoid-rich resin-producing flowers and leaves which, when ingested after heating, have robust, non-lethal, receptor-based effects via the human endogenous cannabinoid, or endocannabinoid, signaling system. Such effects pharmacologically are properly termed “cannabinergic.” The endocannabinoid system is an essential biological signaling system that appeared 600 million years ago in life (Melamede, 2005) and plays a master-regulatory role in many physiological functions that humans may naturally wish to self-adjust, such as mood, appetite, memory, inflammation, muscle tone, pain perception, and stress management, in addition to other more subtle but equally validated functions such as neuroprotection, bone growth, immunity, tumor regulation, seizure threshold, gastrointestinal motility, and intraocular pressure, to name a few (Di Marzo, 2004; Pacher et al., 2006; Vettor et al., 2008).

When gathering evidence to address behavioral questions surrounding human consumption and production of potentially psychoactive cannabis preparations, it is absolutely essential that this long, co-evolutionary arc of human history with this cannabinergic plant be appreciated in order to understand underlying human values, and desires that motivate cannabis use and prevent smokescreen prejudices from taking root. The main question is: what sorts of relationships can humans have with cannabis, aside from aberrant, pathological, and addictive ones? And, as a corollary to this question, when cannabis is consumed in contemporary settings, does it necessarily have to be as a scarce consumerist commodity, or do other relational possibilities exist? By addressing such questions, a richer understanding of cannabis use can emerge and lessen the chance that use patterns are improperly understood as pathological or deviant, when they may fact be perfectly normal and healthful. Certainly the caveat that cultural controls and norms regarding cannabis use that play an important public health role may not translate to all social groups must be acknowledged.

A broader understanding of the human-cannabis relationship beyond the dominating twentieth century American and colonial prohibitionist sociolegal frameworks is needed. When there is not a war against cannabis being fought, a less distorted picture of its effects can emerge. The element of psychological distress that cannabis prohibition regimes produce is worth seriously accounting for as it can play a significant role in the conflation of the effect of cannabis on a user with the effect of the criminal or social stigma attached to that use (Aggarwal et al., 2012). A research approach from social science known as political ecology, taken from anthropology and geography, which is able to incorporate into its analysis the total human-plant relationship and the effects of local and global sociopolitical forces, is helpful here (Robbins, 2004). Political ecology is framework used to study human-environment relations that joins cultural ecology with political economy. Cultural ecology studies how cultural groups adapt, adjust, and relate to their natural environments, and political economy studies how political institutions, the political environment, and economic systems influence each other (Mayer, 1996; Johnston et al., 2007). A sampling of the results of applying such an approach to demystify the smokescreen was given above.

By applying political ecology to cannabis use and production, we can begin to understand and appreciate traditional ecological knowledge regarding its use and production, extant and extinct cultural practices surrounding cannabis use, and the history of their marginalization. Western delegates first heard officially from other countries who wished not to impose absolute prohibition at United Nations meetings in the early 1960s when the first comprehensive international treaty that would call for strict controls on cannabis was being negotiated. Indeed, while a number of thriving civilizations have found a way to integrate cannabis use into their legally sanctioned cultural fabrics, such alternate sociocultural and political realities were ultimately targeted for suppression.

Substantial evidence has been gathered regarding the efficacious use of cannabis as a medicine to treat specific conditions. Additionally, convincing evidence regarding the use of cannabis as a non-problematic “recreational” psychoactive substance with a low potential for addiction has been collected and become increasingly accepted in the US and abroad. Public policy regimes recognizing such use patterns—medical marijuana and adult marijuana use—have taken root in several US states and internationally. However, two human-cannabis use relationships, oft-neglected in medical and public health literature, but for which substantial evidence exists are cannabis use as a *spiritual or religious activity* and as *an herbal or dietary supplement*. These use patterns were presented by international delegates from countries such as India and Pakistan for respectful consideration at the UN but simply ignored and censured (United Nations, 1961; Times of India, 2012). I call for more research and documentation on these use patterns globally using the research framework described to fully eradicate the smokescreen and see clearly what exists.
